# Access to medicines for the management of asthma in Nigeria: is it getting worse?

**DOI:** 10.11604/pamj.2025.52.114.49850

**Published:** 2025-11-17

**Authors:** Kosisochi Chinwendu Amorha, Ogechi Christiana Obi, Chukwudi Richard Ifeanyi, Mmaduabuchi Desmond Udoji, Angela Chinasa Ani, Ruth Nkechi Sabastine, Ifeoma Felicita Uzu, Faith Nnenna Eze, Oluebubechukwu Praise Eze, Chisom Peace Ezeala, Chinelo Cynthia Ezenwafor, Adaora Anthonia Agubata, Oluoma Maryjane Chukwuwa, Chinagozim Rufina Nworah, Emelda Chinemerem Omene, Chidimma Elizabeth Mbakamma, Chisom Sandra Ibenekwu, Somtochi Prosper Nwani, Jennifer Chigozie Iganga, Chidera Edith Eze, Immaculata Chidimma Aniebonam, Kossy Maryann Ochi, Rita Nnenne Oparaocha, Chibueze Raymond Okoye, Kende Kelvin Shima, Adanne Onyedikachi Amorha

**Affiliations:** 1 Department of Clinical Pharmacy and Pharmacy Management, Faculty of Pharmaceutical Sciences, University of Nigeria, Nsukka, Enugu State, Nigeria,; 2 Asthma Awareness and Care Group, Nsukka, Enugu State, Nigeria,; 3 National Institute for Pharmaceutical Research and Development, Abuja, Nigeria,; 4 EHA Clinics, Abuja, Nigeria,; 5 MedPlus Pharmacy Limited, Lagos State, Nigeria,; 6 Department of Clinical Pharmacy and Pharmacy Management, Faculty of Pharmaceutical Sciences, Nnamdi Azikiwe University, Awka, Anambra State, Nigeria,; 7 University of Nebraska at Kearney, Nebraska, United States,; 8 Heartland Alliance Ltd/GTE Community Facility, Benin, Edo State, Nigeria

**Keywords:** Access, affordability, asthma, availability, Nigeria, prices

## Abstract

Persistent inflation, economic instability, limited healthcare resources, and the withdrawal of major pharmaceutical companies from Nigeria have constrained access to affordable asthma medications. This commentary highlights the challenges faced by patients with asthma in obtaining essential medicines for symptom relief and control in Nigeria, while advocating for sustainable strategies to improve access across the population. Several barriers contribute to the unavailability of asthma medicines in pharmacies. Even when available, their costs remain prohibitive for most patients. To address this, medicine prices should be subsidised, local pharmaceutical manufacturers encouraged to produce essential asthma therapies, and health insurance schemes expanded to include and cover these vital medications. This could improve equitable access and outcomes for individuals living with asthma in Nigeria.

## Commentary

A patient experiencing an acute asthma attack was rushed to a hospital. Confidently, the nurse contacted the emergency pharmacy unit for salbutamol nebules, only to hear, “*We don´t have any available*.” This scenario illustrates a recurring reality in many federal and state medical facilities in Nigeria, where over 13 million individuals are estimated to be living with asthma. Despite being underdiagnosed, asthma remains highly prevalent in sub-Saharan Africa, with Nigeria bearing a large portion of the burden [1]. Poor disease management, limited medical resources, inadequate healthcare infrastructure, and the unavailability of essential medicines all contribute to this public health challenge [2]. Beyond the physical and emotional toll of the disease, individuals with asthma in Nigeria face the additional hardship of accessing the recommended medications. These systemic barriers place patients at heightened risk of avoidable morbidity and mortality. This commentary highlights the major obstacles to asthma medicine access in Nigeria and proposes strategies to mitigate them.

**Barriers to asthma medicine access:** medicines listed in the World Health Organization (WHO) Essential Medicines List (EML) are not consistently available across many regions in Nigeria [1]. While drug unavailability is a critical problem, affordability represents an even greater challenge, given the exorbitant prices of anti-asthmatic medications in the current Nigerian pharmaceutical market. Ongoing inflation, prolonged economic crises, and the withdrawal of multinational pharmaceutical companies further threaten access to affordable asthma medications.

**Economic factors:** inflation in Nigeria has greatly compounded the struggles faced by patients with asthma in accessing essential therapies. Although the annual inflation rate reportedly eased from a peak of over 34% in June 2024, it remained above 20% as of August 2025, with food inflation exceeding 21% [3]. Since food expenses constitute a major component of household spending, rising costs often compel individuals to prioritize basic sustenance over healthcare. Given that over 80% of Nigerians pay out-of-pocket for medicines, escalating living expenses translate directly to reduced access to essential drugs. Consequently, healthcare becomes deprioritized, worsening the management of chronic diseases such as asthma. The implications extend beyond individual health. Inflation-induced healthcare inaccessibility hampers productivity and perpetuates the cycle of poverty. With persistent labour market weakness and poor economic growth, the number of people living below the poverty line continues to rise [4]. Many of these individuals inhabit environments that predispose them to ill-health and catastrophic healthcare expenditures [5]. The intersection of economic decline, high living costs, and escalating drug prices forces patients to ration medications or turn to unregulated herbal alternatives, with potentially grave consequences.

**System-level challenges:** Nigeria´s fragile healthcare system further compounds these difficulties. The public health sector, already under-resourced, struggles to maintain a steady supply of affordable essential medicines [6]. Although initiatives such as the National Health Insurance Authority (NHIA) have modestly improved medicine access among enrollees [7], the scheme´s reach remains limited. The majority of Nigerians, particularly those unemployed or in the informal sector, are not covered. A possible reason includes, and is not limited to, ignorance about its existence or how to get registered. Even within accredited facilities, stock-outs of covered medicines are common [7]. As a result, patients often resort to private pharmacies where prices are markedly higher; an untenable situation for low-income populations already burdened by financial hardship [8].

**Pharmaceutical market challenges:** the exodus of multinational pharmaceutical companies has further destabilized Nigeria´s pharmaceutical landscape. In 2023, several global firms, including GlaxoSmithKline (GSK), exited direct operations after over five decades in the country. GSK´s shift to third-party distribution was intended to maintain supply continuity; however, regulatory bottlenecks, weak infrastructure, and currency fluctuations have undermined this approach. The company´s withdrawal, coupled with that of AstraZeneca, makers of Symbicort® inhaler (formoterol/budesonide), has created a substantial gap in the availability of high-quality asthma medications. Essential brands such as Ventolin® (salbutamol) and Seretide® (salmeterol/fluticasone) have become scarce and prohibitively expensive, leading to poor asthma control and increased hospitalizations. Local pharmaceutical companies, while growing, lack the technical capacity and financial incentives to fill this production void. Few, if any, currently include essential asthma medicines in their manufacturing portfolios.

**Outcomes:** the consequences of inadequate asthma management are multifaceted, ranging from recurrent hospital admissions to decreased productivity and diminished quality of life. Limited access to quality-assured medicines has eroded patients´ trust in the healthcare system and fuelled the proliferation of counterfeit and substandard drugs. In an unregulated market, patients may unknowingly purchase ineffective or unsafe products, undermining both clinical outcomes and confidence in pharmacotherapy. The situation is particularly dire for low-income earners, for whom the cost of a single inhaler may exceed a day´s wage. The lack of affordable, effective alternatives not only heightens the risk of uncontrolled asthma but also increases preventable morbidity and mortality [9].

## Conclusion

Nigeria faces an impending asthma crisis unless urgent, coordinated interventions are implemented to restore access to essential medications. The government and relevant stakeholders must prioritize patient-centred strategies to stabilize medicine supply and pricing. These should include providing targeted subsidies for asthma medicines, incentivizing local pharmaceutical manufacturing, and offering research grants to indigenous drug development institutes. Moreover, supportive fiscal and trade policies are needed to address foreign exchange volatility, which directly affects medicine importation and pricing. Expanding national health insurance coverage to include essential asthma medicines would also reduce the financial burden on patients. In the immediate term, subsidizing asthma treatments for individuals within the minimum wage bracket could significantly improve access and outcomes. Without such measures, millions of Nigerians living with asthma will continue to face unnecessary suffering and avoidable deaths from a preventable and manageable condition.
